# Phacoemulsification versus Phacoemulsification/Trabeculectomy for the Treatment of Primary Open-Angle Glaucoma Coexistent with Cataract: A Comparative Study

**DOI:** 10.3390/medicina59030470

**Published:** 2023-02-27

**Authors:** Athanasios Kaliardas, Irini Chatziralli, Andreas Katsanos, George Kitsos

**Affiliations:** 1 Ophthalmology Clinic, University of Ioannina, 45500 Ioannina, Greece; 2 2nd Department of Ophthalmology, University of Athens, 12462 Athens, Greece

**Keywords:** glaucoma, cataract, phacoemulsification, trabeculectomy, treatment

## Abstract

*Background*: The purpose of this study was to evaluate the clinical outcomes in patients with primary open-angle glaucoma (POAG) and coexistent cataract treated with phacoemulsification cataract surgery, either alone or in combination with trabeculectomy. *Methods*: Participants in this retrospective study were 52 patients diagnosed with previously controlled POAG and coexistent cataract, who underwent either uneventful phacoemulsification cataract surgery (Group I, n = 27) or combined uneventful phacoemulsification cataract surgery and trabeculectomy (Group II, n = 25), with at least a 24-month postoperative follow-up. We recorded the changes in intraocular pressure (IOP) and in the need of anti-glaucoma medications before and after surgical procedures. *Results*: There was a statistically significant decrease in IOP at postoperative day 7 in both groups (*p* < 0.001), which remained until the end of the 24-month follow-up. At month 24, the two groups did not differ significantly in terms of IOP (14.3 ± 1.4 vs. 13.1 ± 1.2 for Group I and Group II, respectively; *p* = 0.447). In addition, there was a statistically significant decrease in the number of anti-glaucoma medications needed at postoperative day 7 in both groups (*p* < 0.001 for both groups compared to baseline). At month 24, patients in both groups needed about one additional anti-glaucoma medication to control their IOP. Of note, during the first month after surgery, 20% of patients in Group II needed 0.1 mL 5-FU injections to the bleb, although antimetabolites were not used in the primary surgery. *Conclusions*: Both surgical interventions, namely phacoemulsification cataract surgery alone and phacoemulsification/trabeculectomy, were found to be effective in the management of POAG with coexistent cataract, presenting a significant decrease in IOP and in the need of anti-glaucoma medications postoperatively at a long-term follow-up period of 24 months.

## 1. Introduction

Glaucoma is the leading cause of irreversible blindness worldwide, and it is estimated to affect about 112 million individuals worldwide in 2040 [1,2]. Primary open-angle glaucoma (POAG), which is the most common form of glaucoma, may coexist with cataract [3], especially in adults over 65 years, with about 10% of the elderly with cataract having ocular hypertension or glaucoma [4,5].

Primary therapeutic options for POAG include medical treatment and laser therapy, which have been found to effectively decrease the intraocular pressure (IOP) and prevent glaucoma-related visual loss [6]. In addition, surgical treatment modalities such as trabeculectomy or tube implantation have been used for more severe or recalcitrant cases [7], while the most recent minimally invasive glaucoma surgery devices (MIGS) also provide encouraging results [8].

In fact, trabeculectomy is the most widely performed IOP-lowering procedure, in which a channel between the anterior chamber of the eye and the subconjunctival space is created to allow control of IOP [9]. The procedure can be performed either alone or in combination with cataract surgery to reduce IOP, as well as the medication burden in eyes with POAG coexistent with cataract, although the optimal management of these patients remains controversial. Specifically, removing the lens before trabeculectomy may be a treatment approach in such patients, increasing the anterior chamber depth and reducing the risk of a postoperative shallow anterior chamber [10]. However, potential postoperative IOP spikes may cause damage on the optic nerve; therefore, trabeculectomy is most frequently performed prior to cataract surgery [11]. In addition, performing trabeculectomy in a phakic eye may be challenging due to anatomical reasons, and it may advance cataract progression [12,13]. As a result, the combined procedure of “phacotrabeculectomy” may be a reasonable treatment option in theory, although it obtained a poor reputation in its early years since it provided poorer surgical results than trabeculectomy alone, and it is now a rarely used procedure in many glaucoma centers [14,15]. On the other hand, phacoemulsification cataract surgery alone has been found to achieve IOP reduction in patients with cataract and coexistent POAG [16].

Based on the above, the purpose of this study was to evaluate the clinical outcomes of patients with controlled POAG and coexistent cataract treated with phacoemulsification either alone or combined with trabeculectomy.

## 2. Material and Methods

Participants in this retrospective study were 52 consecutive Caucasian patients diagnosed with POAG and coexistent cataract who were treated with either phacoemulsification cataract surgery or phacoemulsification combined with trabeculectomy at the University Clinic of Ioannina, Ioannina, Greece, between 2013 and 2017. All participants had POAG, which was previously controlled with anti-glaucoma drops and was found to be stable in the last three visits before any surgical intervention. In addition, all patients had uncomplicated surgical procedures and at least a 24-month follow-up after surgery. Patients with secondary glaucoma, such as lens-induced, neovascular, or uveitic glaucoma; a previously failed trabeculectomy; or other intraocular surgeries; were excluded from the study. The study was in adherence with the tenets of Helsinki Declaration and was approved by the institutional review board of the University of Ioannina, Medical School (516/12-10-2020). Written informed consent was obtained from all participants before entering the study.

Before any intervention, all participants underwent a thorough ophthalmological examination, including best-corrected visual acuity measurement by means of Snellen charts, IOP measurement by Goldmann applanation tonometry, gonioscopy, slit-lamp examination and dilated fundoscopy, as well as visual fields testing to confirm the diagnosis of POAG. The demographic characteristics of patients were recorded, along with the maximum and the mean IOP before any surgical procedure, as well as the medications received for the treatment of POAG. No washout was performed prior to measurement of pre-operative IOP. All participants were allocated to two groups, based on the surgical procedure they had undergone: either phacoemulsification cataract surgery alone (Group I, n = 27) or combined phacoemulsification and trabeculectomy (Group II, n = 25). All surgeries were performed by the same surgeon (GK). Intraocular pressure at different time-points (baseline, day 7, month 1, month 6, month 12, and month 24 postoperatively), as well as intra- and postoperative complications, were recorded. As baseline IOP, we defined the mean pre-operative IOP, which was compared with the IOP at the different time-points of the follow-up.

### 2.1. Surgical Procedures

Phacoemulsification cataract surgery in Group I was performed under local anesthesia (tetracaine hydrochloride 1% eye drops) using the Infinity Vision System (Alcon Laboratories, Inc., Fort Worth, TX, USA). A 2.75 mm clear corneal incision and side-port paracentesis were made. A viscoelastic injection was administered, and a continuous curvilinear capsulorhexis was performed with capsulorhexis forceps, followed by hydrodissection, phacoemulsification, irrigation and aspiration of the cortical remnants, and posterior chamber intraocular lens (IOL) implantation. Viscoelastic was subsequently removed and surgical wounds were hydrated with balanced salt solution (BSS). No sutures were needed. All wounds were checked for leakage and found to be watertight.

Phacoemulsification cataract surgery combined with trabeculectomy in Group II was performed under peribulbar anesthesia (5 cc of 2% xylocaine hydrochloride and 2 cc of 0.5% bupivacaine hydrochloride) using 2-site technique. A limbal-based conjunctival flap and a 3.5 mm × 3.5 mm scleral flap were made superonasally with a number 11 blade. Phacoemulsification was performed though a temporal clear corneal incision. Specifically, anterior chamber was entered with a 2.75 mm keratome, and a continuous curvilinear capsulorhexis (5–6 mm) was carried out, followed by hydrodissection, phacoemulsification, cortical aspiration using Alcon Infinity, and implantation of posterior chamber IOL into the bag. The trabeculectomy was subsequently performed by excising a block of 1 × 1 tissue from the posterior lip of the scleral tunnel using a Kelly’s Descemet’s membrane punch. A peripheral iridectomy was performed with Vannas scissors. The scleral flap was then sutured with two 10-0 nylon releasable sutures, and the conjunctiva was closed with a 7-0 Vicryl suture near the limbus in a watertight manner. BSS was injected through the side port into the anterior chamber to observe the formation of diffuse conjunctival bleb and any possible leakage.

Postoperatively, all patients were treated with topical 1% prednisolone acetate six times daily for a week, tapered over 4 weeks (depending on the inflammation); 0.5% moxifloxacin six times and 1% cyclopentolate three times daily for a week were administered to patients of Group II. Patients were evaluated for IOP on postoperative day 7, month 1, month 6, and every 6 months thereafter, with additional visits as and when required.

The primary outcomes were the changes from baseline in IOP and IOP-lowering medications at each postoperative time-point. Safety assessment included the nature, frequency, and timing of adverse events.

### 2.2. Statistical Analysis

A statistical analysis was performed using IBM SPSS Statistics version 23.0. For the description of demographic and clinical characteristics of the study sample, mean and standard deviation (SD) for continuous variables, as well as counts with percentages for categorical variables, were used. The normal distribution of data was checked using the Shapiro–Wilk test. A Student’s *t*-test for independent samples was performed for comparisons between the two groups, while a paired *t*-test was used to compare parameters over time within the same group in the longitudinal analysis. The statistical significance was set to *p* < 0.05.

## 3. Results

Table 1 shows the demographic and clinical characteristics in both groups of our study sample. The mean age of patients was 73.1 ± 3.3 years. A total of 26 out of 52 patients (50%) was male, and the remaining 26 were female (50%).

**Figure 1:**
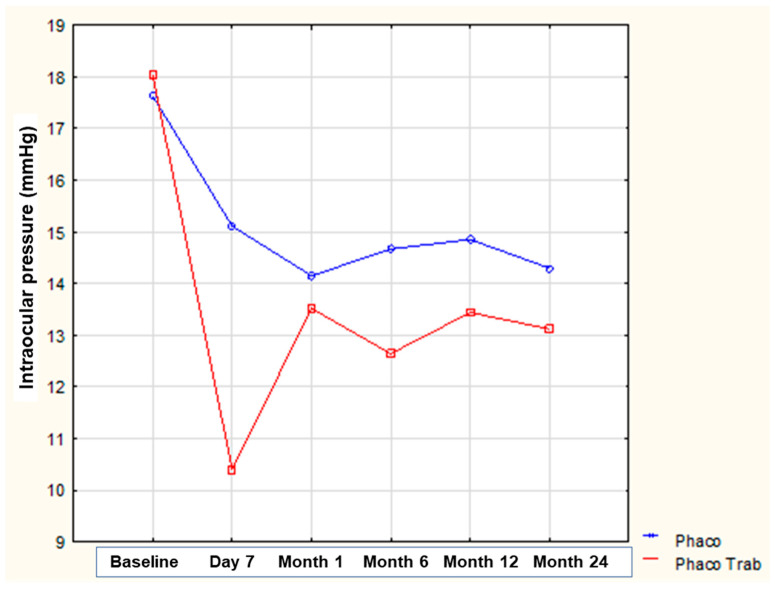
Evolution of intraocular pressure over time between the two groups.

At baseline (before any surgical intervention), the mean IOP was 17.6 ± 1.5 mmHg for the “phacoemulsification” group and 18.0 ± 1.9 mmHg for the “phacoemulsification/trabeculectomy” group, not differing between the two groups (*p* = 0.999). Figure 1 depicts the evolution of IOP over time in both groups. There was a statistically significant decrease in IOP at all time-points in both groups (*p* < 0.001 for all comparisons). Of note, in the “phacoemulsification” group, the IOP decreased between baseline and postoperative day 7, while a relatively stable IOP of about 14.5 mmHg was evident thereafter till the end of the follow-up period. In the “phacoemulsification/trabeculectomy” group, the greater decrease in IOP was observed in the first postoperative week, while the IOP increased significantly at month 1 compared to day 7 postoperatively and then remained almost stable at about 13 mmHg until the end of the follow-up period. Comparing the two groups in between, there was a statistically significant difference in the mean IOP in favor of Group II at day 7 postoperatively (15.1 ± 2.0 vs. 10.4 ± 2.2 for Group I and Group II, respectively; *p* < 0.001) and at month 6 postoperatively (14.7 ± 2.1 vs. 12.7 ± 1.7 for Group I and Group II, respectively; *p* = 0.003), while the two groups did not differ significantly in terms of IOP at postoperative month 1 (14.2 ± 1.6 vs. 13.5 ± 2.7 for Group I and Group II, respectively; *p* = 0.984), month 12 (14.9 ± 1.6 vs. 13.4 ± 1.9 for Group I and Group II, respectively; *p* = 0.176), and month 24 (14.3 ± 1.4 vs. 13.1 ± 1.2 for Group I and Group II, respectively; *p* = 0.447). Table 2 shows the mean change in IOP at different time-points compared to the baseline in both groups.

**Figure 2:**
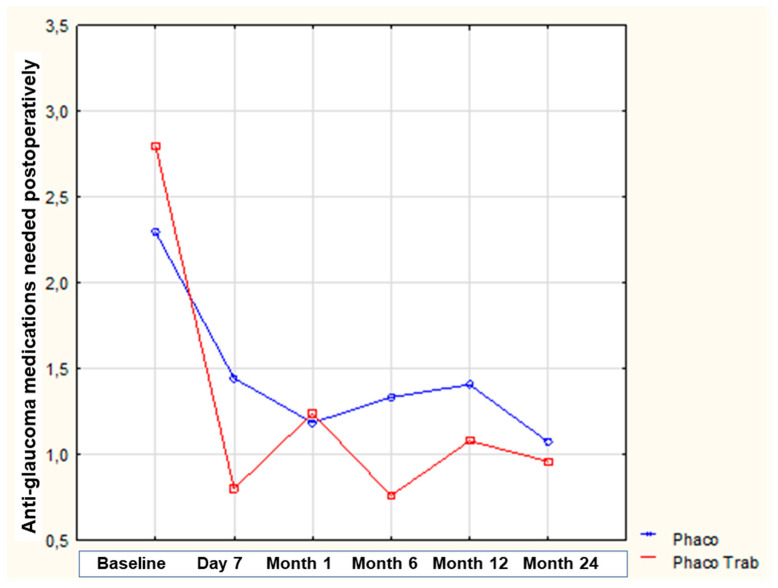
Evolution of anti-glaucoma medications over time between the two groups.

Additionally, there was a statistically significant decrease in the number of anti-glaucoma medications needed at postoperative day 7 in both groups (*p* < 0.001 for all comparisons). At baseline, patients in the “phacoemulsification” group needed on average 2.3 ± 0.8 anti-glaucoma medications/day to control IOP, while those in the “phacoemulsification/trabeculectomy” group received on average 2.8 ± 1.0 anti-glaucoma medications/day. The two groups did not differ significantly regarding the number of anti-glaucoma medications used at baseline (*p* = 0.307). Figure 2 shows the evolution of the number of anti-glaucoma medications needed in both groups over time. It is worthy to note that in both groups, although at the early postoperative period the majority of patients did not use anti-glaucoma medications to control IOP, patients needed about one adjunct anti-glaucoma medication to control their IOP at the end of the 24-month follow-up period. Table 3 presents the changes in the number of anti-glaucoma medications at different time-points compared to the baseline in the two groups. There is a quite significant reduction in anti-glaucoma medications in the “phacoemulsification/trabeculectomy” group at day 7 and months 1, 6, and 12 of the follow-up. However, at month 24 there was no significant difference regarding the change in the number of anti-glaucoma medications used between the two groups, while the difference in the reduction in IOP between the two groups became less at month 24, highlighting the early failure of trabeculectomy in the absence of antimetabolites during surgery.

No significant postoperative complications were recorded in either group. In the first month after surgery, 5 out of 25 patients of Group II (20%) needed adjunct 0.1 mL 5-FU injections above the bleb, although antimetabolites were not used in the primary surgery.

## 4. Discussion

This study demonstrates that both surgical interventions, namely phacoemulsification cataract surgery and combined phacoemulsification/trabeculectomy, effectively lower IOP and the need for IOP-lowering medications in patients with previously controlled POAG and coexistent cataract. Noticeably, at the end of the long-term follow-up of 24 months postoperatively, patients in both groups exhibited a well-controlled IOP of about 13–14 mmHg with only one additional anti-glaucoma medication on average. Both surgical procedures were safe, without reported sight-threatening complications.

Glaucoma and cataract both increase in prevalence with increasing age and frequently coexist. Since elective cataract surgery has been previously shown to decrease IOP in patients with POAG, a reasonable approach to patients with POAG and coexistent cataract could be phacoemulsification cataract surgery alone. On the other hand, the aim of combined surgery in patients with glaucoma and cataract is to reduce IOP and dependency on anti-glaucoma medications with a single procedure.

Previous studies have compared the likelihood of surgical success between phacotrabeculectomy and trabeculectomy, reporting no overall significant difference between the two procedures [9]. However, the criteria used to define complete and qualified success and failure varied considerably among the included studies, making a comparison between studies challenging. In a recent meta-analysis, no difference in postoperative IOP control was found between phacotrabeculectomy and trabeculectomy with or without later phacoemulsification, whereas the complication rate was significantly lower in phacotrabeculectomy [9]. On the contrary, Ogata et al. prospectively reported that phacotrabeculectomy resulted in inadequate IOP reduction in comparison with trabeculectomy alone at year 1 [15]. Consistent with this result, Sacchi et al. retrospectively demonstrated that trabeculectomy alone achieved a higher success rate and lower mean IOP than phacotrabeculectomy for patients with a mean follow-up period of 25.7 ± 14.4 months [17]. These findings could be attributed to the prolonged inflammation after trabeculectomy, which can remain until 6 months after surgery, leading to bleb scarring [18], while flare values return to normal only 4 weeks after phacoemulsification. In addition, phacotrabeculectomy increases the risk of blood–aqueous barrier collapse [19].

On the other hand, when comparing phacotrabeculectomy with small-incision cataract surgery, no difference in the mean IOP reduction, visual acuity, and mean number of anti-glaucoma medication was found between the two procedures, and both appeared to be safe and effective techniques for primary surgery in the patients with coexistent cataract and glaucoma [16]. It is also worthy to note that a drop in IOP has been generally observed in non-glaucomatous eyes with cataract treated with phacoemulsification, suggesting that phacoemulsification may affect IOP, although the exact mechanism remains unclear [20]. In our study, patients with POAG and cataract who underwent “phacoemulsification” and “phacoemulsification/trabeculectomy” both showed a significant decrease in IOP and anti-glaucoma medications. Although at month 6, the “combination” group exhibited better control of IOP, at months 12 and 24, there was no significant difference in the mean IOP between the two groups, suggesting that trabeculectomy may fail, especially in the absence of antimetabolites during surgery.

An interesting point to be mentioned is the time of trabeculectomy in patients with POAG and cataract. Some studies support the synchronous combined phacotrabeculectomy, while other surgeons first perform the trabeculectomy and then the phacoemulsification. In fact, no significant differences in the cumulative probabilities of success were found between the two surgical approaches at a 5-year follow-up period [21]. In our study, we preferred to perform combined phacotrabeculectomy, so as to have the advantage of a single procedure.

Potential limitations of this study pertain to its retrospective design, which did not allow any randomization of patients. However, we tried to minimize the risk of selection bias by including all consecutive eyes undergoing the compared surgical procedures within a specific time frame, not using any pre-existing parameters to allocate a patient to a group. It is worthy to note that the two groups were balanced at baseline in terms of age, gender, IOP, and anti-glaucoma medications used. Furthermore, the study sample was relatively small. A strength of this study is the long-term follow-up period of 24 months, given the chronicity of glaucoma. In addition, the study was comparative, having two arms: one in which patients were treated with phacoemulsification surgery alone and the other in which phacotrabeculectomy was performed. Finally, it is worthy to note that antimetabolites were not used during the initial filtering surgery, although the use of mitomycin seems to be the standard of care with modern trabeculectomy, especially when combined with phacoemulsification.

## 5. Conclusions

In conclusion, our study showed that phacoemulsification cataract surgery, either alone or in combination with trabeculectomy, is a safe and effective procedure to control IOP and to reduce the anti-glaucoma medications burden in patients with previously controlled POAG and coexistent cataract, suggesting that such patients may benefit from phacoemulsification, which is a widely performed surgical procedure. Further prospective randomized studies with large study samples are needed to warrant our results.

## Figures and Tables

**Table 1:** Demographic and clinical characteristics of our study sample.

	Phacoemulsification Group (n = 27)	Phacoemulsification/Trabeculectomy Group (n = 25)	*p* Value
Age (mean ± SD, years)	73.2 ± 3.0	73.0 ± 3.7	0.843
Gender (N, %)			0.999
*Male*	13 (48.1%)	13 (52%)	
*Female*	14 (51.9%)	12 (48%)	
IOP at diagnosis (mean ± SD, mmHg)	29.7 ± 3.8	31.0 ± 4.0	0.235
Maximum IOP preoperatively (mean ± SD, mmHg)	20.8 ± 1.2	22.0 ± 1.5	0.084
Baseline IOP (mean ± SD, mmHg)	17.6 ± 1.5	18.0 ± 1.9	0.999
Number of preoperative anti-glaucoma medications (N, %)			0.751
1	4 (14.8%)	3 (12.0%)	
2	12 (44.4%)	5 (20.0%)	
3	10 (37.0%)	11 (44.0%)	
4	1 (3.7%)	6 (24.0%)	

IOP: intraocular pressure; SD: standard deviation.

**Table 2:** Change in intraocular pressure at different time-points compared to baseline in the two groups.

	Phacoemulsification Group (n = 27)	Phacoemulsification/ Trabeculectomy Group (n = 25)
Postoperative day 7	−2.52 ± 2.26	−7.64 ± 2.63
Postoperative month 1	−3.48 ± 1.95	−4.52 ± 3.48
Postoperative month 6	−2.96 ± 2.39	−5.40 ± 1.04
Postoperative month 12	−2.78 ± 2.01	−4.60 ± 1.22
Postoperative month 24	−3.33 ± 1.82	−4.92 ± 1.80

**Table 3:** Change in the number of anti-glaucoma medications at different time-points compared to the baseline in the two groups.

	Phacoemulsification Group (n = 27)	Phacoemulsification/ Trabeculectomy Group (n = 25)
Postoperative day 7	−0.85 ± 0.72	−2.00 ± 0.58
Postoperative month 1	−1.11 ± 0.70	−1.56 ± 0.65
Postoperative month 6	−0.96 ± 0.71	−2.04 ± 0.61
Postoperative month 12	−0.89 ± 0.64	−1.72 ± 0.68
Postoperative month 24	−1.22 ± 0.93	−1.84 ± 0.69

## Data Availability

Data available upon request.
